# The tumor suppressor phosphatase PP2A-B56α regulates stemness and promotes the initiation of malignancies in a novel murine model

**DOI:** 10.1371/journal.pone.0188910

**Published:** 2017-11-30

**Authors:** Mahnaz Janghorban, Ellen M. Langer, Xiaoyan Wang, Derek Zachman, Colin J. Daniel, Jody Hooper, William H. Fleming, Anupriya Agarwal, Rosalie C. Sears

**Affiliations:** 1 Department of Molecular and Medical Genetics, Oregon Health & Science University, Portland, Oregon, United States of America; 2 Papé Family Pediatric Research Institute, Oregon Stem Cell Center, Department of Pediatrics, Portland, Oregon, United States of America; 3 Department of Pathology, Johns Hopkins University School of Medicine, Baltimore, Maryland, United States of America; 4 Knight Cancer Institute, Oregon Health & Science University, Portland, Oregon, United States of America; 5 Division of Hematology & Medical Oncology, Oregon Health & Science University, Portland, Oregon, United States of America; 6 Brenden-Colson Center for Pancreatic Care, Oregon Health and Science University, Portland, Oregon, United States of America; Katholieke Universiteit Leuven, BELGIUM

## Abstract

Protein phosphatase 2A (PP2A) is a ubiquitously expressed Serine-Threonine phosphatase mediating 30–50% of protein phosphatase activity. PP2A functions as a heterotrimeric complex, with the B subunits directing target specificity to regulate the activity of many key pathways that control cellular phenotypes. PP2A-B56α has been shown to play a tumor suppressor role and to negatively control c-MYC stability and activity. Loss of B56α promotes cellular transformation, likely at least in part through its regulation of c-MYC. Here we report generation of a B56α hypomorph mouse with very low B56α expression that we used to study the physiologic activity of the PP2A-B56α phosphatase. The predominant phenotype we observed in mice with B56α deficiency in the whole body was spontaneous skin lesion formation with hyperproliferation of the epidermis, hair follicles and sebaceous glands. Increased levels of c-MYC phosphorylation on Serine62 and c-MYC activity were observed in the skin lesions of the B56α^hm/hm^ mice. B56α deficiency was found to increase the number of skin stem cells, and consistent with this, papilloma initiation was accelerated in a carcinogenesis model. Further analysis of additional tissues revealed increased inflammation in spleen, liver, lung, and intestinal lymph nodes as well as in the skin lesions, resembling elevated extramedullary hematopoiesis phenotypes in the B56α^hm/hm^ mice. We also observed an increase in the clonogenicity of bone marrow stem cells in B56α^hm/hm^ mice. Overall, this model suggests that B56α is important for stem cells to maintain homeostasis and that B56α loss leading to increased activity of important oncogenes, including c-MYC, can result in aberrant cell growth and increased stem cells that can contribute to the initiation of malignancy.

## Introduction

Protein Phosphatase 2A (PP2A) is a heterotrimeric Serine-Threonine protein phosphatase that is ubiquitously expressed in eukaryotic cells [1] and mediates 30–50% of cellular Serine/Threonine protein phosphatase activity [2]. PP2A is involved in the regulation of numerous signaling pathways, and as such, contributes to stem cell self-renewal, proliferation, differentiation, migration, cell survival, and apoptosis. The PP2A heterotrimeric holoenzyme consists of three major subunits: a catalytic (C) subunit, a structural (A) subunit, and a variable regulatory (B) subunit, which directs the PP2A holoenzyme to a specific target and location [1, 3]. In mammals, the A and C subunits are found in two isoforms, α and β. Aα and Aβ are 87% identical, whereas Cα and Cβ share 97% identity [3]. To date, four unrelated families of B subunits have been identified: B, B′, B″, and B′″ [3, 4]. Altogether, 15 genes encode 26 B subunits of PP2A, which can potentially assemble more than 100 distinct PP2A complexes [3–7]. Many of these subunits have distinct spatial and temporal expression patterns, and details about the different complexes and their activities remain incompletely understood.

PP2A plays an important tumor suppressor role in cell transformation. Previously, an siRNA screen was performed to determine which PP2A regulatory subunits within the PP2A complex were implicated in this process, and this work showed that B56α, B56γ, and PR72/PR130 were the only B subunits critical for regulating human cell transformation in these assays [8]. PP2A complexes containing these B subunits regulate key oncogenic pathways, including c-MYC (MYC), Wnt, and PI3K/AKT signaling [8, 9]. In particular and relevant for this work, PP2A-B56α binds and directly dephosphorylates MYC at a conserved residue, Serine62 (S62), which leads to destabilization of the MYC protein [9].

MYC is a transcription factor that regulates many genes involved in critical cellular functions such as proliferation, growth, and apoptosis and is overexpressed in about 70% of human cancers [10–13]. Loss of regulation of several post-translational modifications of MYC, including phosphorylation at S62 (pS62), results in increased MYC stability and activity contributing to cancer formation [11, 12, 14–17]. Phosphorylation at Threonine 58 (pT58) following S62 phosphorylation has an opposing effect on MYC stability [18]. T58 phosphorylation facilitates PP2A-B56α-mediated dephosphorylation of pS62 and recruitment of the proteasomal degradation complex consisting of the E3 ubiquitin ligase SCF^Fbw7^ [19, 20]. This process is facilitated by the scaffold protein AXIN1, which helps nucleate a destruction complex for MYC at target gene promoters [21, 22].

In addition to MYC, PP2A-B56α also negatively regulates β-catenin, ERK and BCL-2. It has been shown that B56α is part of an AXIN1-mediated degradation complex for β-catenin and B56α overexpression reduces β-catenin expression in mammalian cells and *Xenopus laevis* embryo explants [23]. B56α, through activated type I TGF-β receptor, is recruited to ERK and inactivates it [24]. B56α was also shown to co-localize with BCL-2 at the mitochondrial membrane and suppress its pro-survival activity [2, 25]. Despite the important role of B56α in regulating key oncogenes and cell transformation, only one study has reported a function of B56α *in vivo*, and it was focused on determining the influence of B56α on heart functions [26]. This study showed that PP2A-B56α limits phosphatase activity in the heart and that reduced B56α expression results in conduction defects, slower heart rates, and increased heart rate variability [26].

Here we generated a hypomorphic B56α (B56α^hm/hm^) mouse to characterize B56α’s tumorigenic functions. Although the reduction of B56α was ubiquitous, the primary phenotype we observed with B56α reduction was spontaneous skin lesion formation that occurred in conjunction with an increase in immune cell infiltration in the skin lesions, spleen, liver, lung, and lymph nodes of the intestine (mesenteric LN). The skin lesions showed with hyperproliferation of the epidermis, hair follicles and sebaceous glands as well as increased expression of pS62-MYC and the MYC target, Cdk4. Moreover, the number of skin and bone marrow stem cells was increased in the B56α^hm/hm^ mice, and in a chemical-induced carcinogenesis assay, papilloma initiation was accelerated but progression was not affected. Our model suggests that B56α is important for cellular homeostasis, and B56α loss contributes to aberrant cell proliferation and aberrant stem cell maintenance.

## Results

### Generation of transgenic mice with hypomorphic expression of B56α (B56α^hm/hm^)

**Fig 1:**
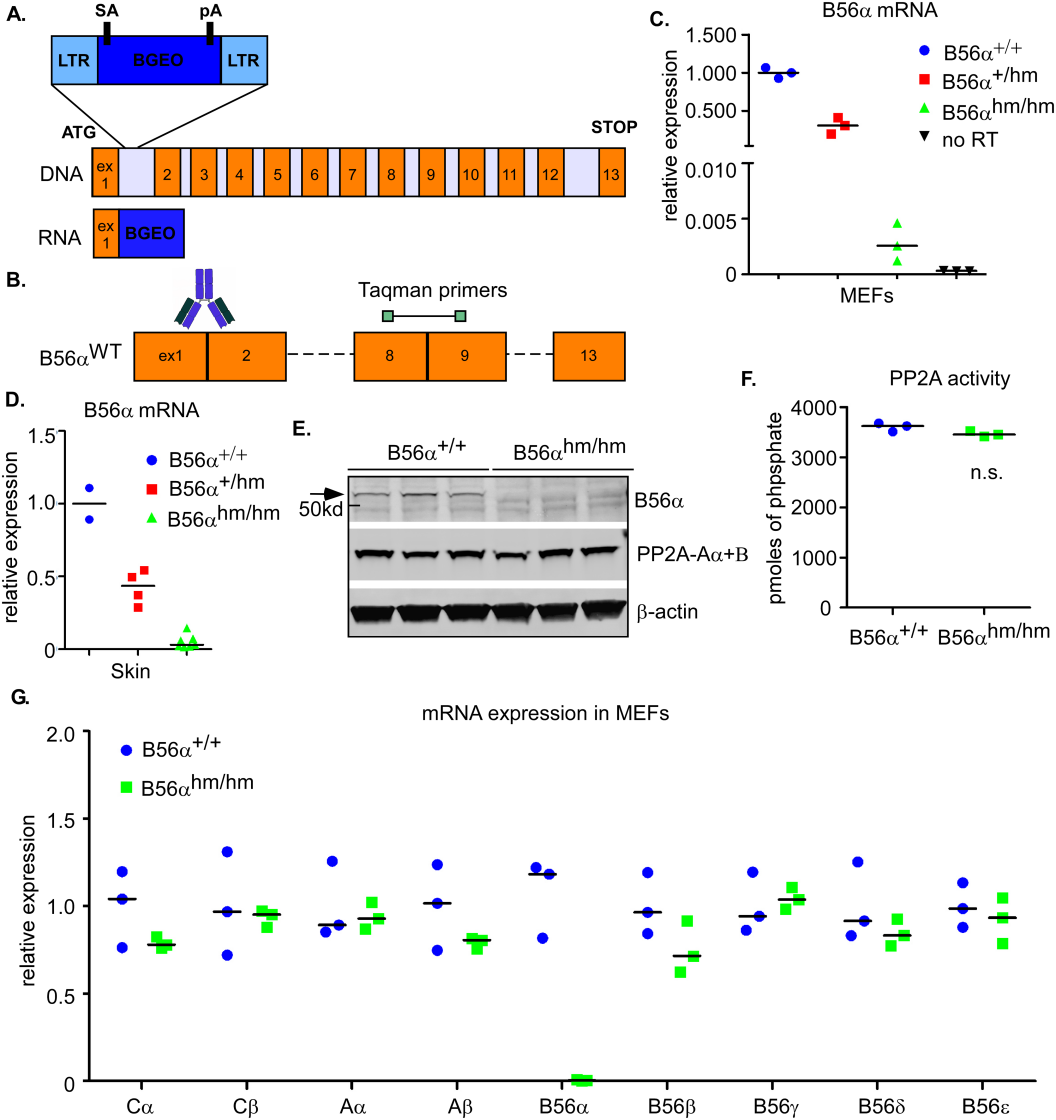
Generation of transgenic mice with hypomorphic expression of B56α. A) A schematic of the *PPP2R5A (B56α)* gene and the gene-trap vector. The vector contains a splice acceptor (SA) site immediately upstream of a promoterless *BGEO (lacZ-neo)* gene. Its integration into intron 1 of B56α leads to expression of a fusion transcript of exon 1 of *B56α* and *BGEO*. B) Schematic of localization of the Taqman qRT-PCR primer set and recognition site for anti-B56α. C) qRT-PCR analysis of B56α mRNA expression in MEFs (n = 3 for each genotype). Relative expression is calculated by ΔCT normalized to the level in B56α^+/+^ MEFs. D) Average of 2 independent qRT-PCR analyses of B56α mRNA expression in skin (n = 2 for B56α^+/+^, n = 4 for B56α^+/hm^, and n = 7 for B56α^hm/hm^). Relative expression is calculated by ΔCT normalized to the level in B56α^+/+^ skins. E) Western blot analysis of MEF (n = 3) lysates for B56α and PP2A-Aα+β. β–actin is a loading control. F) *In vitro* PP2A phosphatase activity of MEFs (n = 3). A two-tailed Student t-test showed no significance. G) qRT-PCR analysis of mRNA expression of different PP2A C, A and B56 subunits in MEFs (n = 3) normalized to the mean level in B56α^+/+^.

Despite an important role for B56α in cell transformation, to our knowledge, the contribution of PP2A-B56α on cell transformation *in vivo* has not yet been determined. PPP2R5A (B56α) deficient mice were generated by integration of a gene trap vector with a splice acceptor (SA) site followed by the *lacZ-neo (BGEO)* cassette (developed by Texas A&M Institute for Genomic Medicine (TIGM)) into the first intron of B56α (Fig 1A) [27]. Mice homozygous for the gene trap allele were born healthy in normal ratios, were fertile, and had no obvious developmental defects. To confirm loss of B56α expression, we performed qRT-PCR and Western blot analysis (see Fig 1B for a schematic of where the primers and the antibody map onto B56α). qRT-PCR analysis on RNA from multiple tissues of 8-week old littermates, from mouse embryonic fibroblasts (MEFs), and from the skin of a separate group of mice showed very low (~0.01–0.2% of control), but persistent expression of B56α in the mice homozygous for the gene trap allele (Figs 1C, 1D and S1A) and, therefore, we refer to these alleles as hypomorphic (hm; B56α^hm/hm^). To test whether bypass of the splice acceptor site in the gene trap or an alternative start site contributed to this low expression of B56α, we designed primers that amplify only exon 1 or from exon 1 to 3, flanking the gene trap insertion (S1B Fig). We determined that the residual mRNA expression of B56α was at least in part due to low-level bypass of the SA since we were able to amplify mRNA using the exon 1 and 3 primer pair (S1C Fig). Western blot analysis revealed reduction of B56α protein expression in B56α^hm/hm^ MEFs (Fig 1E).

Because the B56α^hm/hm^ mice did not show an obvious phenotype at a young age, we sought to determine whether the B56α deficiency was being compensated for by another family member. Therefore, we first asked whether total PP2A activity was changed. Total PP2A activity in MEFs was assessed with an *in vitro* phosphatase assay and was found to not be significantly altered (Fig 1F). To test if B56α deficiency was compensated by altered expression of other subunits, we performed qRT-PCR analysis of all PP2A C, A, and B56 subunits in MEFs, but found no significant changes in the mRNA expression of these subunits (Fig 1G). Moreover, the protein level of the A subunits did not seem to change in the B56α^hm/hm^ mice as compared to wildtype (Fig 1E). Finally, we also performed qRT-PCR for all B56 subunits in skin, thymus, spleen, heart, lung, and liver in B56α^+/+^ and B56α^hm/hm^ mice, but again found no evidence of transcriptional compensation by other family members (S1D Fig).

### B56αdeficient mice develop spontaneous skin lesions

**Fig 2:**
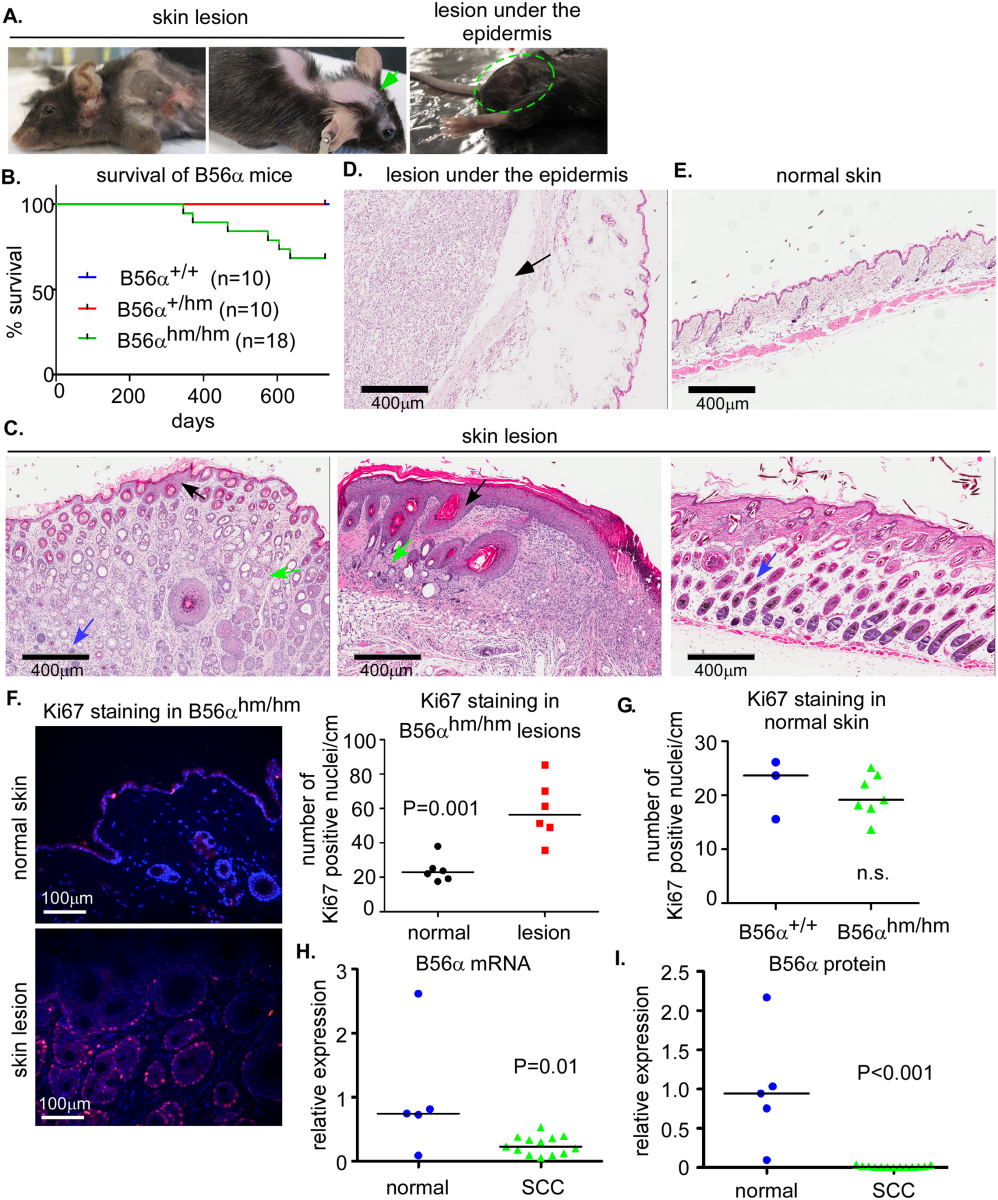
B56α^hm/hm^ mice develop spontaneous lesions. A) Representative pictures of mice with spontaneous skin lesions. One mouse (right panel) had a large mass under the normal skin (lesion under the epidermis, H&E shown in (D)). B) Survival curve of a cohort of B56α mice. C) H&E staining of skin lesions showing hyperproliferation of the epidermis (black arrow), sebaceous glands (green arrow), and hair follicles (blue arrow). D) H&E staining of the lesion under the epidermis. E) H&E staining of wildtype normal skin. F) Quantification of Ki67 staining in B56α^hm/hm^ normal skin and matched lesions (n = 6) in 1cm section of skin. Representative images are shown. p-value is from a two-tailed Student t-test. G) Quantification of Ki67 staining in normal skin of B56α^+/+^ (n = 3) and B56α^hm/hm^ (n = 7) mice in 1cm section of skin. H) Graph showing expression of B56α mRNA in normal (n = 5) human skin versus SCC (n = 13) from patient samples as determined by qRT-PCR. The median and p value from a two-tailed Student t-test is shown. I). Graph showing expression of B56α protein in normal (n = 5) human skin versus SCC (n = 13) from patient samples as determined by Western blot analysis and normalized to GAPDH. The median and p value from a two-tailed Student t-test is shown (Western blot is shown in S3A Fig).

To determine the physiologic function of B56α, a cohort of B56α^hm/hm^ and wild type mice were aged for 22 months and their phenotypes are summarized in Table 1. Approximately 33% of mice (6 out of 18) in the B56α^hm/hm^ cohort were euthanized before 22 months because of poor body condition. The primary phenotype we observed in these mice was spontaneous skin lesion formation with hair loss (Fig 2A). We observed that 28% (5/18) of the B56α^hm/hm^ mice developed skin lesions between 11 to 21 months of age (Fig 2A, 2B and 2C). The mice sacrificed early also had either enlarged liver, and/or spleen, and/or lymph node around the intestine that will be discussed in more detail below. One mouse (1/18) that did not have a skin lesion instead had a 2cm benign non-lymphoid mass of unknown origin under the epidermal layer (Fig 2A; right, and Fig 2D). One mouse with a skin lesion also had a liver tumor that upon histologic analysis appeared undifferentiated (S2A Fig). The rest of the B56α^hm/hm^ mice (n = 12) showed no obvious phenotype and appeared healthy to the study’s endpoint, at which point we performed additional analyses on these and the control wildtype mice (n = 10). We found that some of the B56α^hm/hm^ mice at study endpoint had enlarged spleen or enlarged lymph nodes around their intestine. Two mice that appeared healthy had also lost hair on the back, and upon histologic analysis were found to have a pre-malignant skin phenotype (S2B Fig). All of the wildtype mice, upon histologic analysis of the skin and other organs, appeared normal (Figs 2E and S2B).

**Table 1:** Summary of B56α^hm/hm^ phenotypes in mice with early skin lesions or at the study endpoint.

	Early (6 of 18 mice)	Endpoint (12 of 18 mice)	Total (18 mice)
**Skin lesions**	5 of 18 (28%)	2 of 18 (11%)	7 of 18 (39%)
**Tumor under epidermis**	1 of 18 (6%)	0 of 18 (0%)	1 of 18 (6%)
**Liver tumor**	1 of 18 (6%)	0 of 18 (0%)	1 of 18 (6%)
**Enlarged liver**	2 of 18 (11%)	1 of 18 (6%)	3 of 18 (17%)
**Enlarged spleen**	3 of 18 (17%)	1 of 18 (6%)	4 of 18 (22%)
**Enlarged mesenteric LN**	1 of 18 (6%)	2 of 18 (11%)	3 of 18 (17%)

Table showing number of lesions present in mice that displayed skin lesions early (spontaneously occurring before study endpoint) or that were assessed at the study endpoint (22 months). Percentages indicate the frequency of each lesion (early, endpoint, or total) out of the total number of mice analyzed (n = 18).

H&E staining of all skin lesions showed hyperproliferation of the epidermis and dermis including the hair follicles and sebaceous glands (Figs 2C and S2B). Immunofluorescence (IF) staining for Ki67 in the skin lesions showed increased cell cycling as compared to normal skin (Fig 2F). In the normal skin, however the number of proliferative cells was not different between the two genotypes (Fig 2G). Consistent with this, *ex vivo* B56α^hm/hm^ MEFs also did not show a dramatic difference in proliferation or apoptosis over time (S2C Fig). This might be explained by potential compensatory mechanisms in the absence of an oncogenic event that result in equal total PP2A activity (see Fig 1F) and that are independent of changes in mRNA expression of the B56 subunits (see Figs 1G and S1D).

Because of the observed skin lesion phenotype, we wanted to determine whether B56α expression was altered in human skin lesions. qRT-PCR analysis and Western blot analysis was performed to determine mRNA and protein levels in a set of 5 normal and 13 human squamous cell carcinoma (SCC) samples. We found B56α mRNA and protein expression to be significantly decreased in human SCC tissue as compared to normal tissue (Figs 2H, 2I and S3A). qRT-PCR in a separate set of samples that included SCC as well as many additional types of human skin lesions showed that once again, B56α levels were decreased in many of these lesions (S3B Fig). Together, these results show that loss of B56α often occurs in human skin lesions, which is consistent with a decrease of this protein contributing to transformation of these cells in our mouse model. Although we considered that the prominence of a skin lesion phenotype might indicate differential expression of B56α in this organ, the Human Protein Atlas (www.proteinatlas.org) [28] indicated that the expression of B56α mRNA was similar across many human tissues including skin.

### pS62-MYC is increased in B56α^hm/hm^ skin lesions

**Fig 3:**
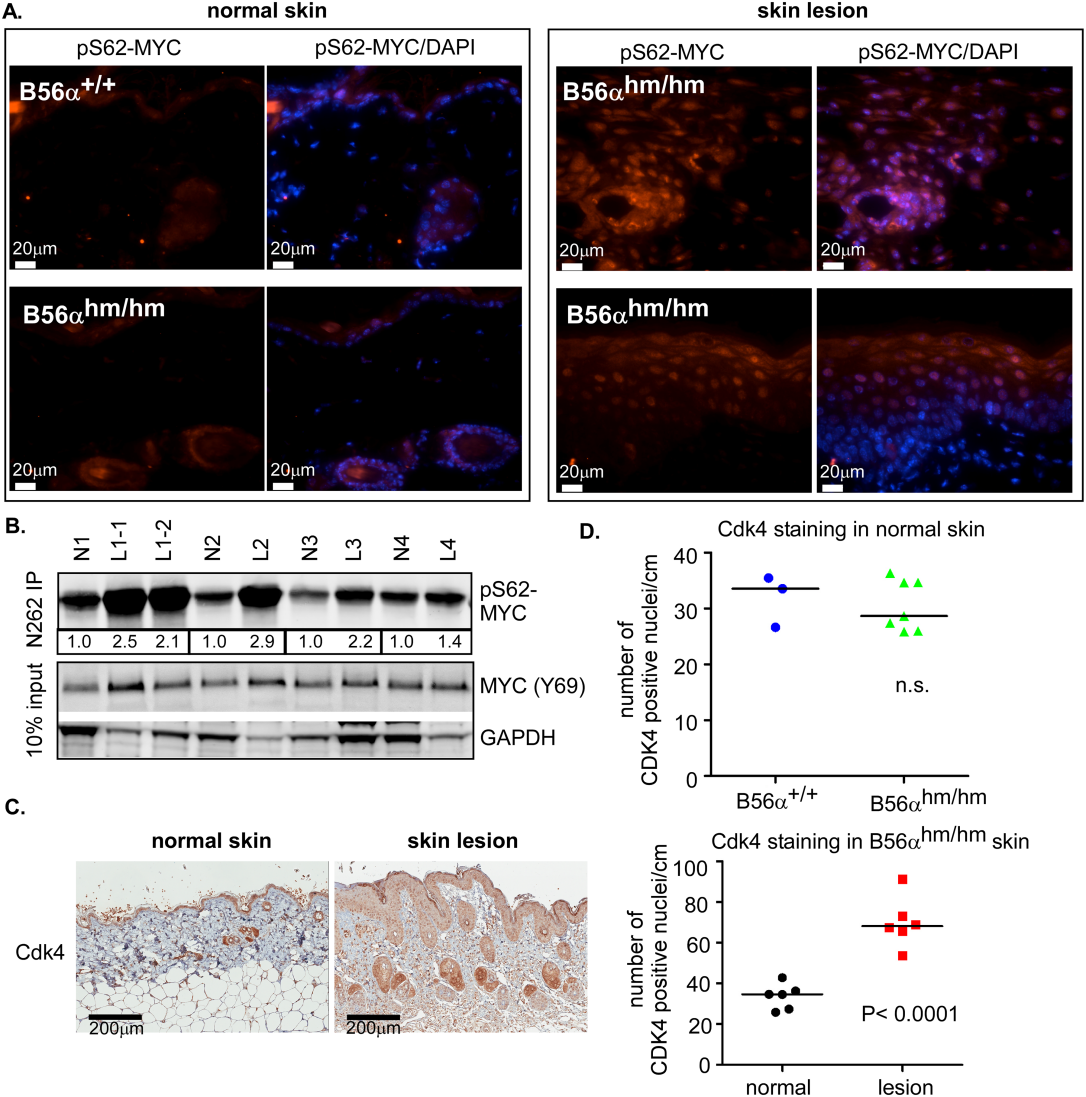
pS62-MYC is increased in B56α^hm/hm^ skin lesions. A) pS62-MYC staining (red) of B56α^+/+^ and B56α^hm/hm^ normal skin and B56α^hm/hm^ skin lesions. DAPI (blue) is a nuclear counterstain. B) pS62-MYC was assessed in B56α^hm/hm^ normal skin and matched skin lesions by an immunoprecipitation (IP)-Western Blot. The bands for pS62-MYC were quantified and values for each lesion relative to its matched normal are displayed below the IP-Western blot. N1 had two lesions (Fig 2A; left) C) Representative IHC images (left) and quantification of Cdk4 positive nuclei (right) in B56α^hm/hm^ normal skin and skin lesions (n = 6). Quantification of nuclear Cdk4 staining in 1cm section of skin was done using the Aperio ImageScope software 11.2.0.780 (Aperio Technologies). The median and p-value from a two-tailed Student t-test is shown. D) Quantification as in (C) of Cdk4 positive nuclei in 1cm section of skin in normal B56α^+/+^ (n = 3) and B56α^hm/hm^ (n = 7) skin.

To further analyze the skin lesions that occurred in our B56α^hm/hm^ mice, we assessed the level of pS62-MYC in the normal skin and skin lesions since PP2A-B56α has been shown to dephosphorylate MYC at this residue. IF analysis showed that pS62-MYC was increased in the skin lesions of B56α^hm/hm^ mice when compared to the normal skin of B56α^hm/hm^ or B56α^+/+^ mice (Fig 3A). The increase in pS62-MYC in B56α^hm/hm^ lesions was quantified by immunoprecipitation of MYC followed by a Western blot for S62 phosphorylation from lysates of normal skin or skin lesions of B56α^hm/hm^ mice (Fig 3B). Consistent with increased activity of MYC in these lesions, we also found that the MYC target Cdk4 was upregulated in the B56α^hm/hm^ skin lesions compared to the normal skin of B56α^hm/hm^ mice (Fig 3C), but that there was no difference in Cdk4 levels in the normal skin of B56α^+/+^ and B56α^hm/hm^ mice (Fig 3D). Finally, we assessed the protein level of pS62-MYC with an IP-Western blot from lysates of normal skin and spleen and a Western blot from lysates of lung and heart and found no difference in the levels of pS62-MYC in these normal tissues from B56α^+/+^ versus B56α^hm/hm^ mice (S4A and S4B Fig). Together, these data indicate that MYC activity is not increased in the normal tissue of the B56α^hm/hm^ mice, but that it is increased in proliferative skin lesions that arise in response to B56α deficiency.

### B56α deficiency induces stemness and promotes initiation of skin papillomas in mice

**Fig 4:**
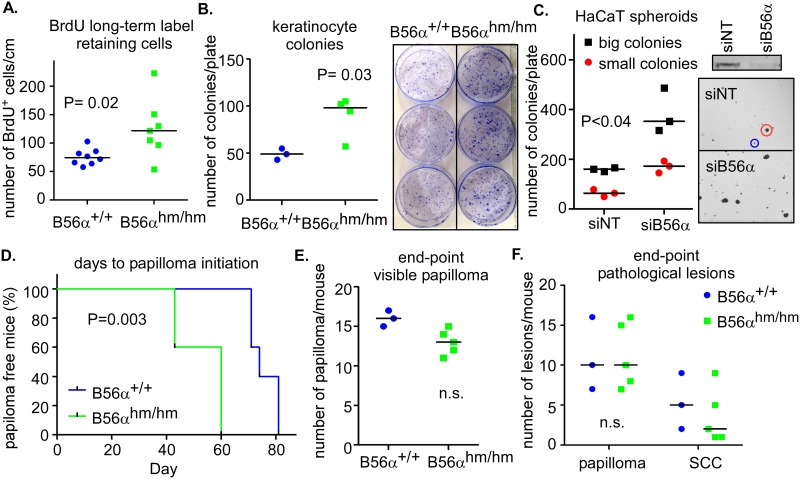
B56α deficiency induces stemness and promotes initiation of skin papillomas in mice. A) Total number of BrdU long-term label retaining cells/cm of skin section in a cohort of mice (n = 8 per genotype). B) Number of colonies per plate from fast-adhering colony forming assay for keratinocyte stem cells (n = 3 for B56α^+/+^ and n = 4 for B56α^hm/hm^). Representative pictures are shown. C) Number of small and large colonies per plate present after 7 day colony forming assay with HaCaT cells transfected with siRNA targeting B56α or a non-targeting control (siNT) from 3 replicates. On the right is a Western blot showing expression of B56α in these cells as well as images of colonies with representative large (red) and small (blue) colonies circled. D) Average days until papilloma initiation following two-stage DMBA/TPA chemical carcinogenesis (n = 5 for B56α^+/+^ and n = 5 for B56α^hm/hm^). p-value is from a Log-rank (Mantel-Cox) test. E) Number of visible papillomas per mouse after 20 weeks of TPA treatment (n = 3 for B56α^+/+^ and n = 5 for B56α^hm/hm^). F) Total number of papillomas and SCC conversion per mouse as determined by histopathology of tissue fixed at end-point (n = 3 for B56α^+/+^ and n = 5 for B56α^hm/hm^).

A previous skin model of MYC (K14.*Myc2*) showed that overexpression of MYC results in skin hyperproliferation and enlarged sebaceous glands, suggesting that MYC plays a role in the maintenance of stem cells in the skin [29, 30]. Because we observed similar phenotypes in the B56α^hm/hm^ mice (see Fig 2C), we asked whether B56α deficiency affected stem cell populations. To test this, we performed a Bromodeoxyuridine (BrdU) long-term label-retaining assay in the skin of mice. BrdU incorporates into the DNA of highly proliferative cells during the neonatal time period and after a chase period, only the slow-cycling stem cells remain labeled [31]. We injected 10 day olds pups with BrdU, harvested dorsal skin tissues after 75 days, and found that B56α deficiency increased the number of BrdU long-term label-retaining cells (LRCs) both in the basal layer and follicular bulge (Fig 4A). We confirmed this result by performing a culture of rapidly adherent epidermal cells, which are thought to be equivalent to the label-retaining stem cells [32], using keratinocytes isolated from 3-day-old pups. We found an increased number of rapidly adherent keratinocytes in B56α^hm/hm^ as compared to B56α^+/+^ mice [Fig 4B]. In addition, we knocked down B56α in an immortalized human epidermal keratinocyte cell line, HaCaT [33], and measured the ability of these cells to form spheroids in ultra-low attachment plates containing stem cell media. We found that B56α knockdown significantly increased the number of spheres formed as compared to that of control cells transfected with non-targeted siRNAs (Fig 4C). Altogether, these data suggest that B56α deficiency contributes to increased stemness of cells in the skin of these mice.

Because of the phenotypic similarity between our model and the K14.*Myc2* model, we next asked whether B56α deficiency, like overexpression of MYC in the K14.*Myc2* model, contributes to cell transformation of the skin epidermis using the DMBA/TPA chemical carcinogenesis assay. DMBA initiates tumor formation by inducing mutations in critical genes such as Ha-Ras within stem cells in the bulge region of hair follicles or the basal compartment of the interfollicular epidermis. TPA then promotes tumor growth by altering gene expression and inducing inflammation to drive expansion of the initiated stem cell population [34]. For this experiment, we first backcrossed the B56α mice to an isogenic FVB background as they should have a more uniform response and this background has been reported to be more sensitive to tumor promotion by TPA as well as more susceptible to squamous cell carcinoma (SCC) development [34]. The DMBA/TPA treatment of a cohort of mice showed earlier formation of skin papillomas in the B56α^hm/hm^ mice (Fig 4D). Papillomas formed on average 53.2 days after initiating TPA treatment in B56α^hm/hm^ mice, whereas they appeared 75.6 days after TPA treatment in the wild type mice. After 20 weeks of TPA treatment, however, the total number of papillomas was counted, and no significant difference in the total number of papillomas or the number of papillomas that converted to SCC was found between the two groups (Fig 4E and 4F). We analyzed pS62-MYC expression in the end stage papillomas collected from this study. We did not observe a significant difference in pS62-MYC staining between B56α^+/+^ and B56α^hm/hm^ mice (S4C Fig). This is consistent with the similar rates of papilloma progression we observed between B56α^+/+^ and B56α^hm/hm^ mice. Together, these results suggest that B56α deficiency plays an important role in initiation, but that at least in this model, reduction of B56α does not affect tumor progression.

### Lymphoid expansion as well as increased circulation and colonogenic potential of c-Kit+ stem cells in B56α^hm/hm^ mice

**Fig 5:**
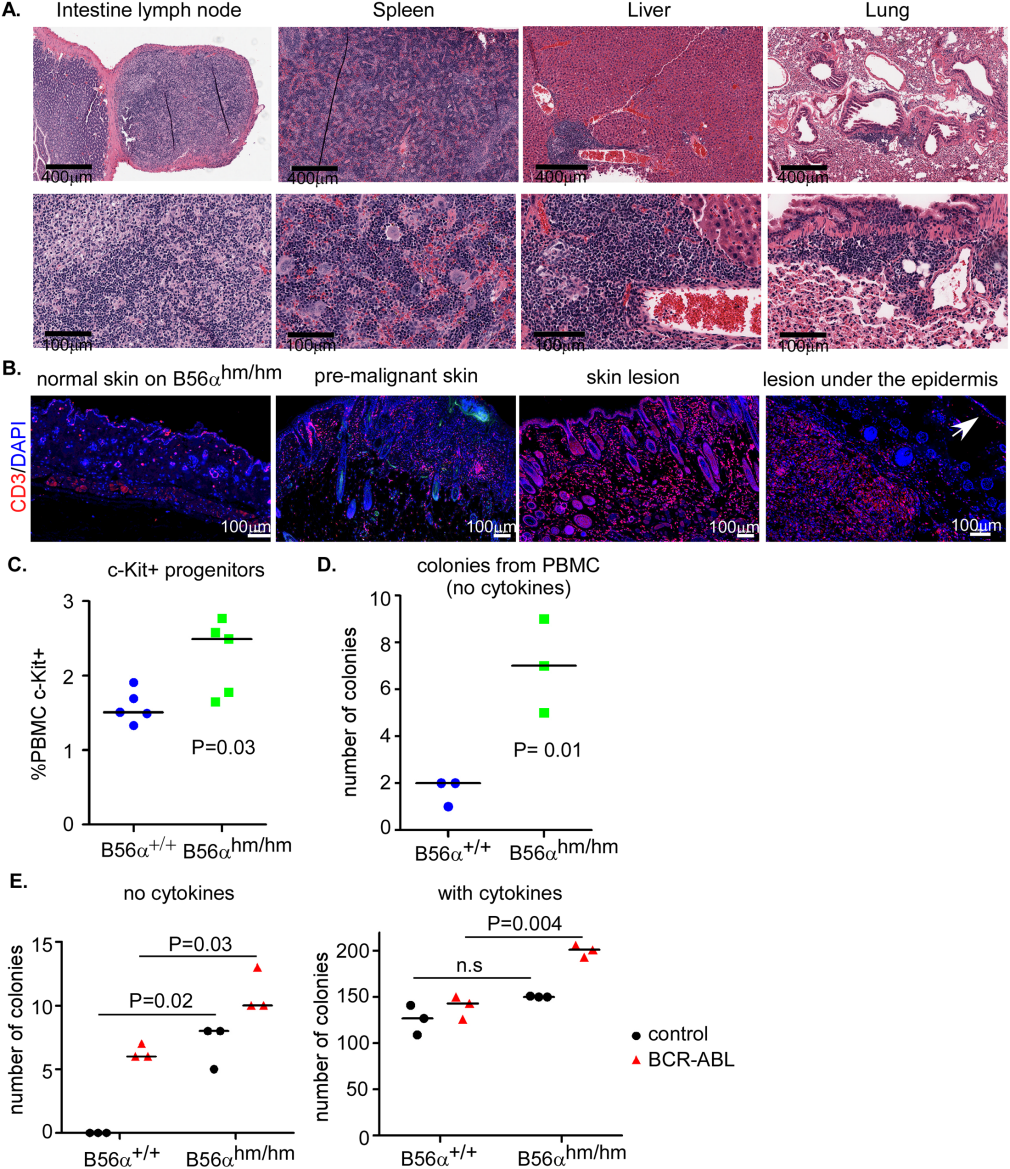
Lymphoid expansion as well as increased circulation and colonogenic potential of c-Kit+ stem cells in B56α^hm/hm^ mice. A) H&E staining shows inflammation in intestinal lymph node, spleen, liver, and lung of mice with skin lesions. B) CD3 staining of normal skin, premalignant skin (normal macroscopically), and skin lesions. On the lesion under the epidermis, the arrow points to normal epidermis, CD3 positive staining is observed in the lesion. C) Flow cytometry for c-Kit^+^ (n = 5 per genotype) and D) CFU assay (in MethoCult, without serum) of isolated PBMCs after injection of mice with GM-CSF. E) CFU assay of bone marrow cells harvested from mice with the indicated genotypes. Cells were infected twice with viruses and plated in MethoCult with and without cytokines in triplicates. Two independent assays were performed. p-value is from a two-tailed Student t-test.

Upon necropsy analysis of mice with skin lesions, we found that some of the mice had an enlarged liver, spleen, or intestinal lymph nodes (see Table 1). H&E analysis of these tissues in addition to lung tissues showed high levels of immune cell infiltrations (Fig 5A). Immunofluorescent analysis of skin lesions as well as the lesion under the epidermis showed increased CD3+ T cells in the lesions as compared to normal skin (Fig 5B). Interestingly, the skin sample that looked macroscopically normal but was premalignant by H&E (shown in S2B Fig) had an intermediate level of CD3 staining suggesting that immune cell infiltration happens early as these lesions start to form (Fig 5B).

Because of the observed increased immune cell infiltration in the peripheral lymph tissues such as spleen, intestine, and liver, we assessed the number and types of mature immune cells (B cells, T cells, and myeloid cells) in the peripheral blood mononuclear cells (PBMCs) by flow cytometry at baseline or in response to GM-CSF addition [35]. For a baseline measurement, blood was harvested from 6-week old mice and analyzed by flow cytometry. There was no difference in the number or type of immune cells found in peripheral blood between B56α^+/+^ and B56α^hm/hm^ mice (S5A Fig). We next asked whether this was altered following a stimulus of four GM-CSF injections, but again found no difference in the number of circulating mature immune cells between wild-type and B56α^hm/hm^ mice (S5B Fig).

One explanation for the increased immune cell infiltration observed in B56α^hm/hm^ mice with lesions could be extra-medullary hematopoiesis, hematopoiesis outside of the medulla of bone. Extra-medullary hematopoiesis presents as hematopoietic masses in various body locations, typically in the spleen and liver, but also can occur in lymph nodes, thymus, heart, breasts, prostate, broad ligaments, kidneys, skin, peripheral and cranial nerves, and the spinal canal [36]. If this were contributing to the lymphoid expansion, we would expect increased circulation of c-Kit+ stem cells in the PBMC following a stimulus. Therefore, we quantified the c-Kit^+^ circulating stem cells in PBMC following four GM-CSF injections and found that B56α^hm/hm^ mice had a 66% increase in the number of c-Kit^+^ circulating cells after stimulation with GM-CSF (Fig 5C). In another approach to quantify circulating hematopoietic stem cells, we performed a Colony-Forming Unit (CFU) assay in the absence of cytokines using whole white blood cells from 6-week old GM-CSF induced mice and found significantly more colonies formed from B56α^hm/hm^ blood cells as compared to that from wild type mice (Fig 5D). These results, along with the histology of lymphoid expansion in multiple tissues, are consistent with possible extra medullary hematopoiesis.

Finally, we tested the ability of bone marrow derived cells harvested from wild type or B56α^hm/hm^ mice to form colonies in the presence or absence of cytokines. Bone marrow derived cells isolated from 6-week old mice were infected with a BCR-ABL expressing retrovirus as a positive control or with an empty vector control retrovirus and then were tested for growth in a CFU assay in the presence or absence of cytokines (described in [37]). We found that cells from B56α^hm/hm^ bone marrow formed significantly more colonies as compared to those from B56α^+/+^ mice in the absence of cytokines, with the number of colonies present similar to that seen with overexpression of BCR-ABL in wild-type cells (Fig 5E). This suggests that reduction of B56α may render these cells more able to grow in the absence of cytokines. In addition, while in the presence of cytokines both B56α^+/+^ and B56α^hm/hm^ cells were able to form colonies, interestingly, only the B56α^hm/hm^ cells showed increased colonogenic capabilities with expression of BCR-ABL under this condition, suggesting that reduction of B56α may cooperate with BCR-ABL for colony formation (Fig 5E). This, together with the GM-CSF stimulation experiments, suggests that the hematopoietic cells in B56α^hm/hm^ mice are normal but that hematopoietic stem cells have increased potential of leaving the niche and forming colonies if they are stimulated resulting in extra-medullary hematopoiesis.

## Discussion

B56α plays an important role in regulating PP2A’s function toward key oncoproteins such as MYC, β-catenin, and Bcl-2. In this study we report that B56α depletion in mice contributes to the formation of skin lesions that show hyperproliferation in the epidermis, hair follicles, and sebaceous glands. Some of the phenotypes we observed, including the skin lesions and hair loss, resembled the phenotypes of previously published K14.*Myc2* mice [29], consistent with the B56α deficiency increasing MYC stability. The K14.*Myc2* mice were shown to gradually lose hair and develop spontaneous ulcerated lesions, which resulted from severe impairment in wound healing. Similar to what we found in B56α^-/-^ skin lesions, K14.*Myc2* skin lesions had hyperproliferative and enlarged sebaceous glands [29]. When we analyzed the B56α^hm/hm^ skin lesions, we observed that pS62-MYC and its target gene Cdk4 were elevated in the lesions. Normal skin in B56α^hm/hm^ mice, however, did not have obvious increased pS62-MYC or Cdk4, suggesting that some additional trigger, potentially mechanical stress or inflammation, results in activation of pathways that lead to an increased pS62-MYC that is maintained at high levels in the absence of B56α.

Previous studies of B56 family protein deficiency, including the *in vivo* loss of B56α, B56γ or B56δ subunits, have shown mild and localized phenotypes with limited compensatory mechanisms suggesting redundancy of either B subunit targeting or the overall control of the PP2A complex activity [26, 38, 39]. Redundancy in the activity of the regulatory subunits has been reported in numerous cellular mechanisms including in chromosome segregation [40], cell survival and development [41], β-Catenin signaling [23, 42, 43], and AKT and ERK activation [8, 44–46], where loss of more than one of the B subunits is needed to observe a more robust phenotype. Here, we find that in normal tissues, as well as in cultured MEFs, hypomorphic deficiency of B56α does not result in an obvious phenotype, as shown by equal proliferation, pS62-MYC levels, Cdk4 levels, and total PP2A activity. We did, however, observe differences in the number of stem cells in the skin and following either aging or chemical carcinogenesis, an increased ability or rate of forming hyperproliferative lesions. To address possible compensation by other B56 regulatory subunits, we measured mRNA expression of these subunits in both MEFs and normal tissues from B56α^+/+^ and B56α^hm/hm^ mice and found no difference at the level of mRNA expression. While this data shows that there is not transcriptional compensation for B56α deficiency by other B56 family members, we cannot rule out the possibility of compensation at the protein level or compensation by other components of the PP2A machinery.

The complexity and mild phenotypes observed in models of knockout of just one B subunit might also be explained by each B subunit having multiple targets. For example, when K14.*Myc2* mice were exposed to chemical (DMBA/TPA) carcinogens, they developed tumors earlier than control mice that ultimately represented a diverse spectrum of lesions including papillomas, SCC, and sebaceous adenomas [30]. In the B56α^hm/hm^ mice, DMBA/TPA treatment also induced earlier development of papillomas, but in contrast to the K14.*Myc2* mice, no difference in total endpoint number of lesions or progression of lesions was observed. One possibility for the absence of increased progression could be the negative regulation of p53 by B56α [47], which could result in p53 becoming active in the B56α^hm/hm^ papillomas and functioning to suppress further transformation.

Previous work has shown that the hair follicular bulge retains stem cells that can be a source for both the normal hair cycle and sebaceous gland renewal. Ectopic expression of *Myc* (*c-Myc-ER-TM*) in the suprabasal epithelial layers of the epidermis and hair follicle has been shown to result in desynchronization of the hair growth cycle associated with a marked increase in cell proliferation along the length of the hair outer root sheath [48]. In addition to MYC, aberrant β-catenin activation, which is another target of B56α, has been implicated in *de novo* hair follicle formation and hair tumors [49]. Consistently, both hair follicles and sebaceous glands were increased in our skin lesions, suggesting a role for B56α in maintaining the stem cells within the follicular bulge. Because of this as well as recent evidence suggesting that PP2A activity gradually increases during the course of human embryonic stem cell (ESC) differentiation [50] and that PP2A-B56α regulates many key factors that are important for stem cell self-renewal including Wnt/β-catenin and MYC, we analyzed whether the number of stem cells in skin was altered in these mice. We found increased stem cells within the skin of B56α mice, both in the basal layer and follicular bulge, in contrast to that seen in the K14.*Myc2* mice, which had a 75% reduction in the number of skin stem cells [29]. Two potential contributing factors to these different results include, 1) high level MYC expression as driven by the *K14* promoter may induce apoptosis in stem cells as has been previously demonstrated [29] while high level of pS62-MYC as driven by B56α deficiency may not [14], or 2) B56α also negatively regulates β-catenin, which is important for stem cell self-renewal. Future experiments will be needed to fully elucidate the role of B56α in the processes controlling self-renewal and maintenance within skin stem cells.

Although the skin lesions that developed in the B56α^hm/hm^ mice were benign, the mice had to be sacrificed because of poor body conditions. The majority of mice with skin lesions were found to have an increased immune cell infiltration in peripheral tissues. B56α mRNA has been shown to be higher in thymus and bone marrow compared to spleen, brain and heart of 10- to 12-week-old C57Bl6 mice [51] and PP2A activation- inhibits AKT and NFκB which results in dampening of T cell proliferation [52–54]. However, we do not find any change in T and B cell populations under normal conditions. Upon skin lesion formation and even pre-lesions, however, we find an increase in immune cell infiltration, particularly T cells. Interestingly, it has recently been shown that PP2A is necessary for normal function of T regulatory (T reg) cells and ablation of PP2A in T regs results in severe multi-organ lymphoproliferative autoimmune disease [55]. Moreover, the mice with T reg-specific PP2A deletion showed dermatitis and in some occasions overt skin rash and ulcerations, as well as lymphoproliferative syndrome with secondary lymphoid organ enlargement including in spleen and mesenteric lymph nodes [55]. This is very similar to the phenotypes we see in our model which suggests that B56α may be important in the regulation of T regs.

The increase in immune cell infiltration, together with the increased number of c-Kit+ stem cells in circulation following GM-CSF stimulation, suggests that B56α deficiency may increase the ability of immune stem cells to migrate out from bone marrow and infiltrate other organs and colonize. Of note, the GM-CSF stimulation experiments that showed increased migration and colonization of immune cells were performed on 6-week old mice, before the generation of skin lesions, so we believe that the immune phenotypes are independent of lesion formation. The immune phenotypes observed may help explain the presence of what appeared to be extra medullary hematopoiesis in these mice.

While B56α has multiple targets, the observed effects in the immune system are consistent with an upregulation of MYC. In previous work, MYC has been shown to control the balance between hematopoietic stem cell (HSC) self-renewal and differentiation in the bone marrow [56]. Normally, HSCs exist in proximity to osteoclasts within the bone marrow, and expression of MYC is required for cells to migrate out of their bone marrow niche to become transient amplifying cells. MYC is then downregulated prior to full differentiation [56].

Furthermore, in the absence of cytokines, reduction of B56α was sufficient to enhance clonogenicity of bone marrow cells and it co-operated with BCR-ABL to further increase the clonogenic potential of these cells. Moreover, while BCR-ABL is not able to increase further colony formation ability of WT bone marrow cells in the presence of cytokines, the reduction of B56α expression allowed BCR-ABL to promote more colony growth. This is consistent with evidence that in blast crisis CML patients, PP2A activity was shown to be impaired due to the overexpression of the PP2A cellular antagonist, SET [57, 58]. Re-activation of PP2A led to a decrease in phosphorylation of various important oncogenic signaling factors including MYC [57]. Further work will be necessary to determine whether B56α plays a non-redundant role in CML or whether loss of other B56 family members would give similar results.

In summary, we report a novel mouse model of B56α deficiency and show that, although B56α may not be required for normal development, it is important for normal homeostasis of stem cells. The primary phenotype observed in these mice is spontaneous skin lesions. They also have increased immune cell infiltrations suggestive of extramedullary hematopoiesis, and inflammation in many organs. Whether increased inflammation contributes to the skin lesions or is caused by skin lesions remains to be understood but lymphocytic inflammation occurred early in the initiation of skin lesions. Increased pS62-MYC and MYC activity is one of the consequences of B56α deficiency. Our model suggests that B56α is important for stem cells to maintain their normal homeostasis and B56α loss can lead to increased activity of important oncogenes such as MYC, contributing to aberrant cell proliferation and loss of stem cell regulation that can support the initiation of malignancy.

## Materials and methods

### Ethics statement

Mice were handled in accordance with the recommendations in the Guide for the Care and Use of Laboratory Animals of the National Institutes of Health. The protocol was approved by the Oregon Health and Science University Institutional Animal Care and Use Committee (IACUC#IS00003989). All mice were housed in the specific pathogen-free (SFP) environment under controlled light cycle, and fed a standard rodent Lab Chow (#5001 PMI Nutrition International) plus water ad libitum.

De-identified human skin lesion and normal samples were provided by OHSU Department of Dermatology Molecular Profiling Tissue Repository (IRB#10071). The NC3Rs ARRIVE guidelines checklist is listed in the S1 Checklist.

### Homozygous B56α gene trap (B56α^hm/hm^) mice

The B56α^hm/hm^ mice (pure C57BL/6N background) were generated from embryonic stem (ES) cell clones carrying a gene trap vector in the *PPP2R5A (B56*α*)* gene obtained from the Texas A&M Institute for Genomic Medicine (TIGM) [27]. The FVB mice were obtained from Jackson laboratory.

### Cell population expansion and apoptosis assay

MEFs isolated from mouse embryos 13.5 days post-coitum were cultured in DMEM+10% FBS. The cell population expansion assay and cytotoxicity (CellTox Green Cytotoxicity Assay, Promega) were performed over 72 hours at passage 1 and passage 8 on an IncuCyte Zoom (Essen Bioscience), which captured images to measure live cell content change over time along with dying cells that are labeled green, in real-time.

### Cell culture, knockdown, and sphere formation assay

The sphere formation assay was performed using HaCaT cells transfected with siB56α and non-targeting siRNAs. Transient knockdown in HaCaT was performed using siRNAs to B56α (L-009352-00-0005) and DharmaFECT1 transfection reagents according to the protocol provided (Dharmacon). Non-targeting siRNA (NTsiRNA) (D-001206-14) was used as a control. After 48 hours, 25,000 cells were plated in ultra-low attachment plates in stem cell media (KnockOut^™^ ESC/iPSC Media Kit (ThermoFisher A1412901), non-essential amino acids (NEAA), and β-Mercaptoethanol (ThermoFisher 21985023)). Total number of spheres was counted after 10 days using the EVOS FL cell imaging system (Advanced Microscopy Group).

### Quantitative RT-PCR (qRT-PCR)

RNA was isolated from homogenized mouse or human tissue using TRIzol reagent (Invitrogen) and cDNA was made as described previously [11]. qRT-PCR was then performed using TaqMan primers for human B56α (Hs00196542-m1), mouse B56α (Mm00523125-m1), or human GAPDH (Hs02786624-g1). The relative fold change was measured by the ΔΔ(C_T_) method described previously [22]. The sequences of additional primers used for RT-PCR are listed in the S1 Table.

### Antibodies for western blot, immunofluorescence (IF), and immuno-histochemistry (IHC)

For IP-Western blots, 350μg of protein from lysates was used, and MYC was pulled down with the N262 antibody (sc-764; 2μg/sample). Western blot analysis was performed as described previously [21]. Immunoblots were visualized using the Odyssey IR imager (LI-COR) that can detect both Fluor 680 and IRDye 800 secondary antibodies (1:10000). Additional antibodies used for western blot include: monoclonal pS62-MYC (1:500, Abcam), Total MYC (Y69) (1:1000, ab32072, Abcam), B56α (1:500, ab72028, Abcam), β-Actin (1:10000, A5441, Sigma) and GAPDH (1:10000, AM4300, Ambion).

Mouse tissues were collected and fixed in 10% formalin-neutral buffer. Paraffin embedding and Hematoxylin and Eosin (H&E) staining were performed at OHSU Histopathology Core. Antibodies used for IF or IHC: pS62-MYC antibody (1:100) we developed as previously described [11] and pS62-MYC (1:500, ab185656, Abcam), Ki67 antibody (1:1000, Novocastra, NCL-Ki67-MM1), anti-BrdU (1:200, MCA2060, AbD serotec), Cdk4 (1:50, sc-260, Santa Cruz Biotechnology), and CD3 (1:100, Dako). IHC images were scanned and quantified by Aperio ImageScope 11.2.0.780 (Aperio Technologies). Antibodies used for the Flow Cytometry assay was described previously [35].

### *In vitro* PP2A phosphatase activity assay

Total PP2A activity was measured by using an *in vitro* colorimetric PP2A phosphatase activity assay (Ser/Thr Phosphatase Assay Kit, Millipore) according to the manufacturer’s protocol. Briefly, lysates from MEFs of B56α^+/+^ and B56α^hm/hm^ mice were prepared in the lysis buffer with low endogenous phosphate (20mM imidazole-HCl, 2mM EDTA, 2mM EGTA, pH 7.0, with 10μg/ml each of aprotinin, leupeptin, antipain, soybean trypsin inhibitor, 1mM benzamidine, and 1mM PMSF). Equal amounts of total protein were used to pull down the PP2A C subunit. The PP2A C activity was then measured by its ability to dephosphorylate the Threonine on the phosphopeptide K-R-pT-I-R-R. The phosphatase release was determined by comparing the absorbance to the standard curve explained in the manufacture’s protocol.

### Two-stage DMBA/TPA chemical carcinogenesis

The back skin of seven-week old mice was shaved 2 days prior to treatment with dimethylbenzanthracene (DMBA, D3254, Sigma-Aldrich, 100μg in 100μl acetone per mouse) [34]. Mice were treated with DMBA once. After 7 days, mice were again shaved and treated with 12-O-tetradecanoylphorbol-13-acetate (TPA, P-1680, LC Laboratories, 2.5μg in 100μl acetone per mouse) twice a week for twenty weeks. Mice were evaluated twice a week for papilloma development. At the end of TPA treatment (20 weeks) all skin papillomas were collected and fixed in 10% formalin-neutral buffer.

### BrdU long-term label retaining cell analysis

Newborn pups (10 days old; n = 8 for each genotype) were injected with 50μl BrdU (Invitrogen) every twelve hours for a total of four injections. Tissue sections (1 cm long) from skin were then collected 75 days after the injection and fixed in 10% formalin-neutral buffer. IF analysis was performed using anti-BrdU and total number of BrdU-labeled cells were counted from the whole section from each mouse.

### Rapidly adherent keratinocyte assay

A rapidly adherent keratinocyte assay was performed as described [32] on cells isolated from B56α^+/+^ and B56α^hm/hm^ mice. Briefly, keratinocytes were isolated from 3-day old pups and 3x10^5^ cells were plated for 10 min at room temperature on dishes coated with collagen type IV. Non-adherent cells were then rinsed off, and rapidly adherent cells were cultured for 20 days in the mouse keratinocyte CnT-07 medium (CELLnTEC). Colonies were stained with crystal violet and counted.

### Immune cell analysis and Colony-Forming Unit (CFU) assay

For PBMC assays, B56α^+/+^ and B56α^hm/hm^ mice were treated with and without GM-CSF as described [35]. PBMCs were isolated and assessed by flow cytometry for B cells (B220), T cells (CD3) and myeloid cells (Mac1/Gr1) as described [35]. Following GM-CSF stimulus, PBMCs were also used to assess the c-Kit+ population by flow cytometry and used in a CFU assay in the absence of cytokines as described [37].

The bone marrow CFU assays were also performed as described [37]. Briefly, bone marrow cells were harvested from B56α^+/+^ and B56α^hm/hm^ mice. Cells were infected with p210^BCR-ABL^ or control retroviruses (described in [37]) two times over 48 hours and then plated in triplicate in MethoCult H3231 (Stem Cell Technologies, Vancouver, BC, Canada) with or without cytokines [37]. Colonies were counted after one week of incubation at 37°C in 5% CO2.

### Statistics

All graphs represent data from three independent experiments (unless otherwise stated in the figure legend) and were generated using GraphPad Prism 5. The median and p value from a two-tailed Student t-test is shown (unless otherwise stated in the figure legend).

## Supporting information

S1 Fig Hypomorphic expression of B56α in different tissues. A) qRT-PCR analysis of B56α mRNA expression in different tissues. All mice with three genotypes are siblings (n = 2 for each genotype). Relative expression is calculated by ΔCT normalized to wild-type B56α. B) Schematic of RT-PCR primers. C) RT-PCR analysis of exon1-exon1 and exon1-exon3 transcripts in different tissues and MEFs from three genotypes. D) qRT-PCR analysis of mRNA expression of different B56 subunits in different tissues normalized to TBP and graphed relative to B56α^+/+^ (total number of mice: B56α^+/+^ = 2 and B56α^hm/hm^ = 3).
(TIF)

S2 Fig Liver tumor in a mouse with skin lesion. A) H&E staining of the liver from a mouse with skin lesion and liver tumor. B) H&E staining of skin from mice at the study endpoint. While all wild type mice have normal skin, two B56α^hm/hm^ mice that were macroscopically normal had pre-malignant lesions. C) Population expansion and apoptosis analysis of MEFs (n = 3 for each genotype) over 72 hours after 1 or 8 passages using live cell imaging and IncuCyte analysis software. Two-tailed Student t-test showed no significant differences.
(TIF)

S3 Fig Expression of B56α is decreased in human skin cancer. A) Western blot of B56α protein expression in 5 normal and 13 SCC patient samples that are quantified in Fig 2I. B) qRT-PCR analysis of B56α mRNA expression in different skin lesions graphed relative to one of the normal skin samples. BCC: Basal Cell Carcinoma, DP: Dermatofibrosarcoma Protuberans, MCC: Merkel Cell Carcinoma, MC: Mucinous Carcinoma, SK: Seborrheic Keratosis, Spindle CC: Spindle Cell Carcinoma.
(TIF)

S4 Fig No difference in c-MYC phosphorylation in different tissues of B56α^hm/hm^ mice. A) IP-Western of pS62-MYC from normal skin and spleen of B56α^+/+^ and B56α^hm/hm^ mice. B) Western blot of pS62-MYC from normal lung and heart of B56α^+/+^ and B56α^hm/hm^ mice. C) IF representative image of pS62-MYC staining (red; ab185656) of B56α^+/+^ and B56α^hm/hm^ DMBA/TPA end stage papilloma lesions. DAPI (blue) is a nuclear counterstain.
(TIF)

S5 Fig No difference in circulating immune cells. A) Flow cytometry for B cells (B220), T cells (CD3) and myeloid cells (Mac1/Gr1) within PBMCs from peripheral blood at the baseline level (n = 3 for each genotype) and after four injections with GM-CSF (n = 5 for each genotype).
(TIF)

S1 Table List of primers designed to amplify exon1-1 and exon1-3 of mouse B56α from cDNA. (PDF)

S1 Checklist The NC3Rs ARRIVE guidelines checklist. (PDF)

## Abbreviations

PP2A: protein phosphatase 2A
SCC: squamous cell carcinoma
