# Supplementary figures and images for: The tumor suppressor phosphatase PP2A-B56α regulates stemness and promotes the initiation of malignancies in a novel murine model
Source: PLoS One. 2017 Nov 30;12(11):e0188910. doi: 10.1371/journal.pone.0188910 (PMC5708644; doi:10.1371/journal.pone.0188910)

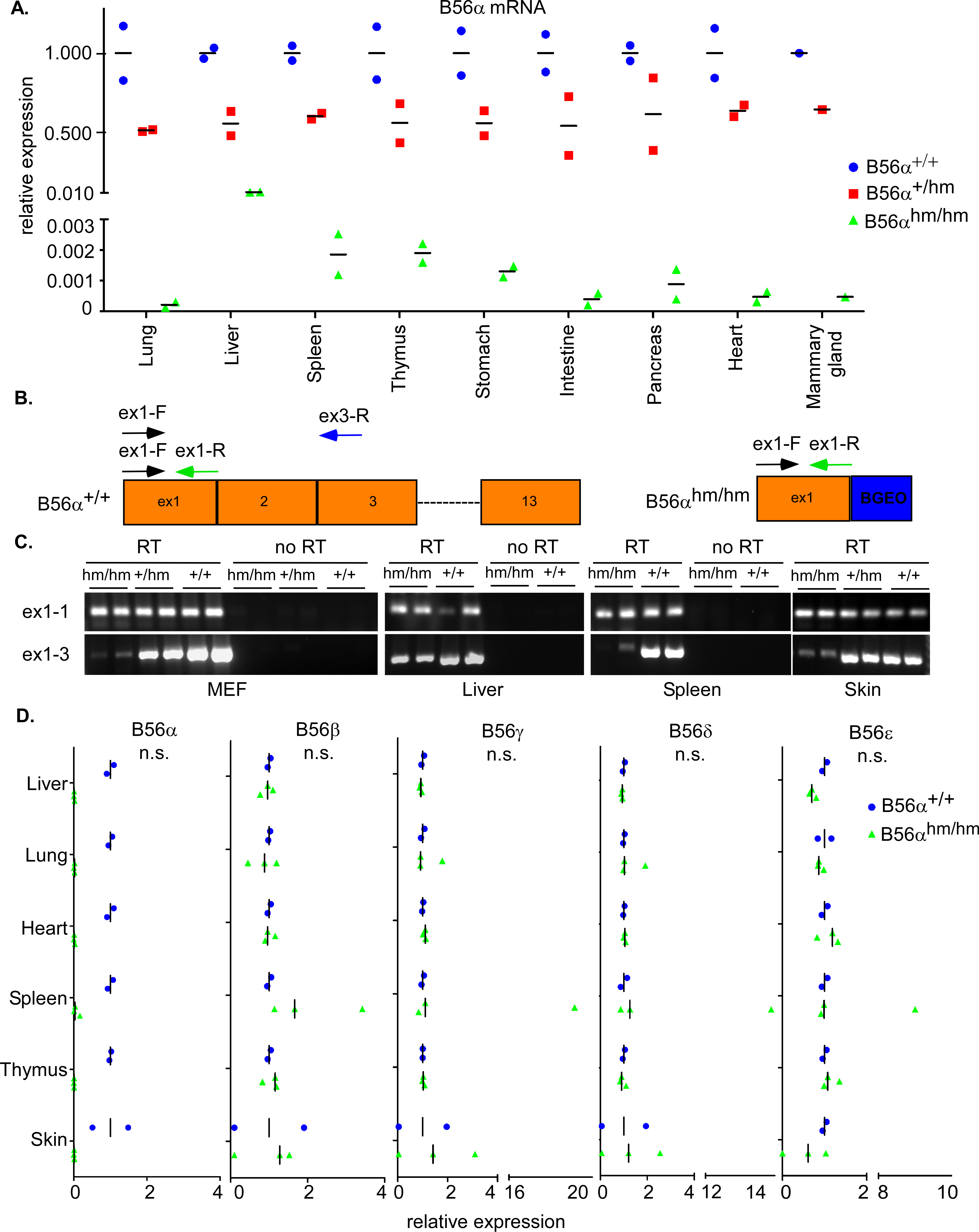

Supplement: S1 Fig — A) qRT-PCR analysis of B56α mRNA expression in different tissues. All mice with three genotypes are siblings (n = 2 for each genotype). Relative expression is calculated by ΔCT normalized to wild-type B56α. B) Schematic of RT-PCR primers. C) RT-PCR analysis of exon1-exon1 and exon1-exon3 transcripts in different tissues and MEFs from three genotypes. D) qRT-PCR analysis of mRNA expression of different B56 subunits in different tissues normalized to TBP and graphed relative to B56α+/+ (total number of mice: B56α+/+ = 2 and B56αhm/hm = 3). (TIF) [file pone.0188910.s001.tif]

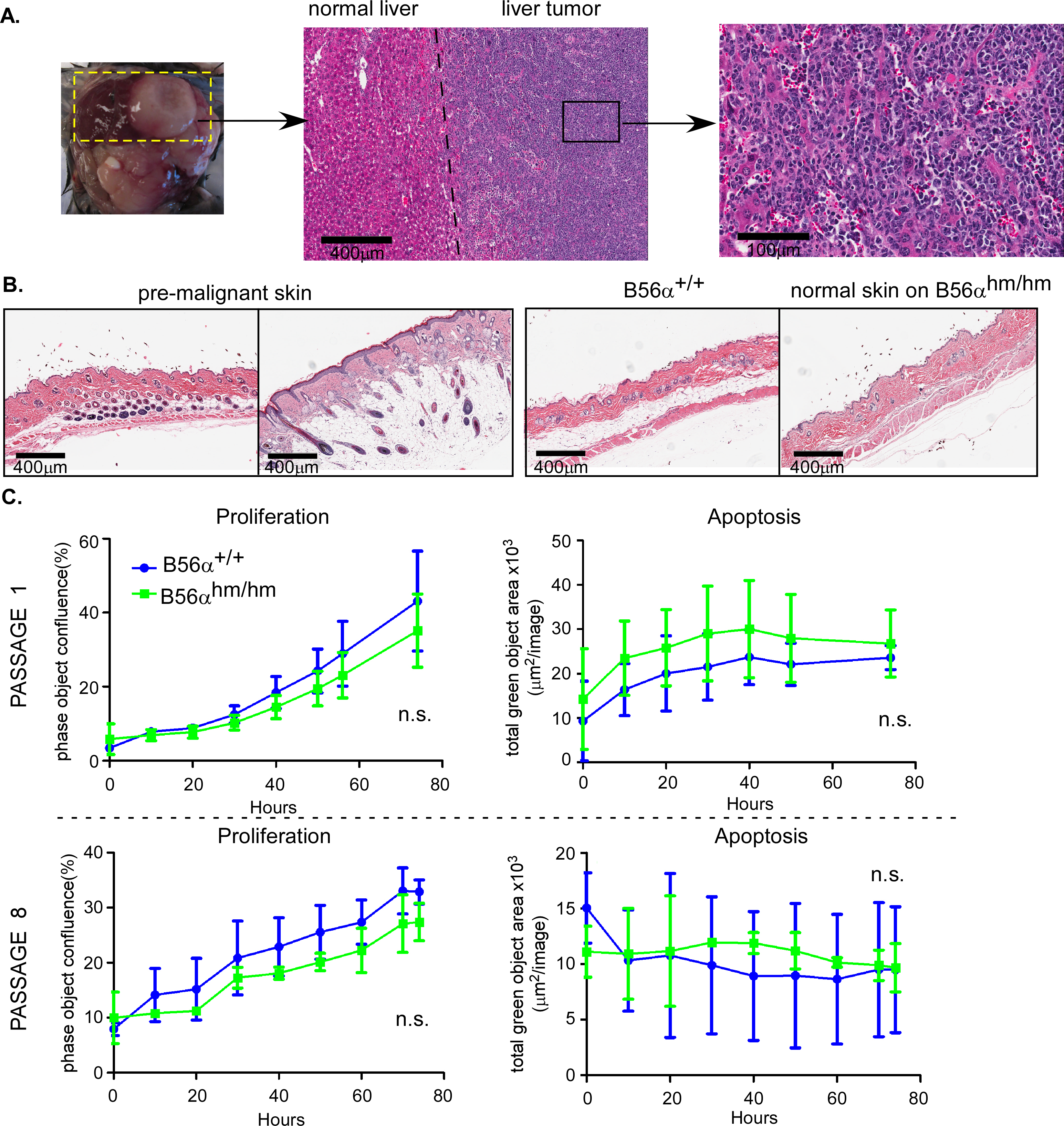

Supplement: S2 Fig — A) H&E staining of the liver from a mouse with skin lesion and liver tumor. B) H&E staining of skin from mice at the study endpoint. While all wild type mice have normal skin, two B56αhm/hm mice that were macroscopically normal had pre-malignant lesions. C) Population expansion and apoptosis analysis of MEFs (n = 3 for each genotype) over 72 hours after 1 or 8 passages using live cell imaging and IncuCyte analysis software. Two-tailed Student t-test showed no significant differences. (TIF) [file pone.0188910.s002.tif]

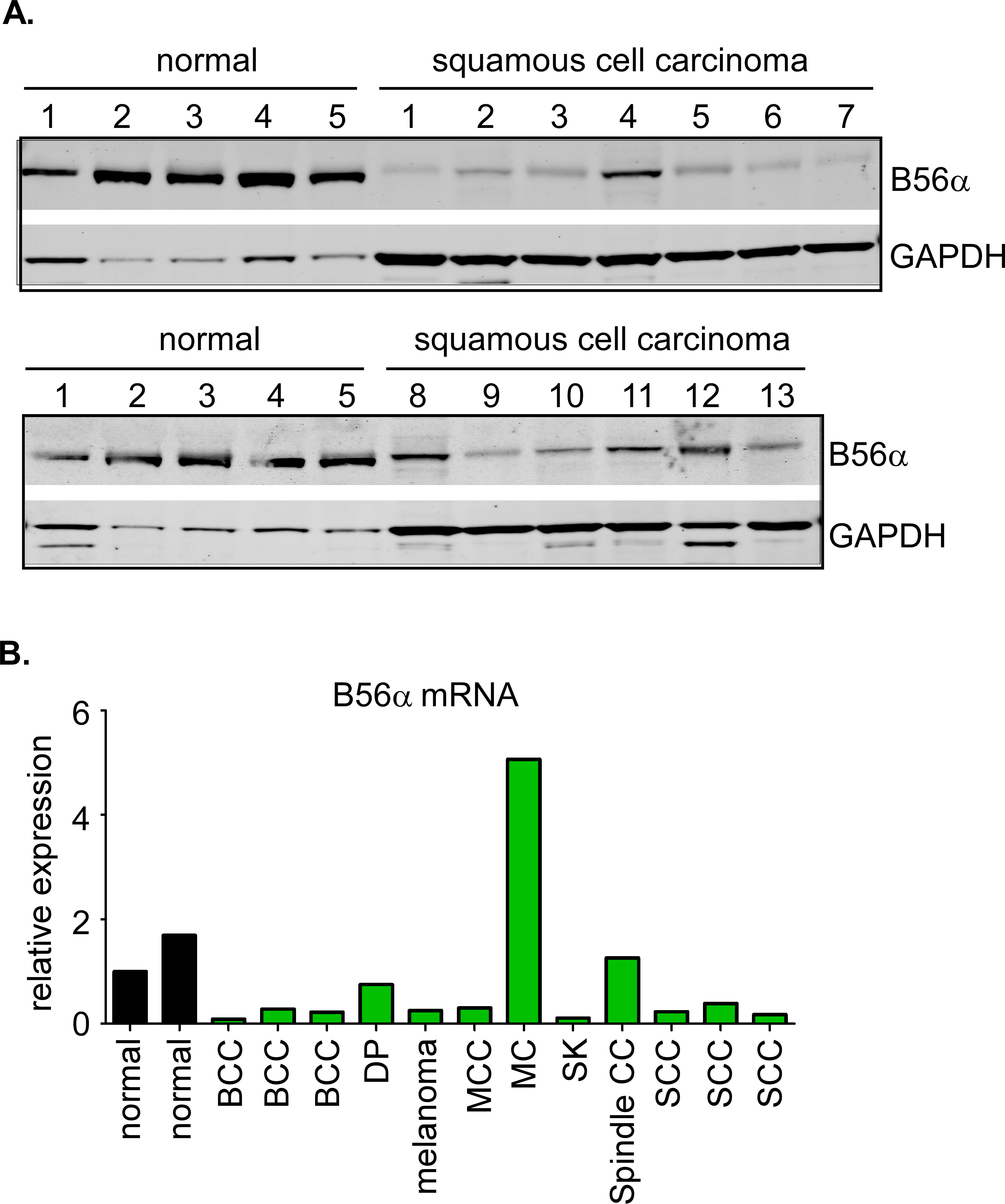

Supplement: S3 Fig — A) Western blot of B56α protein expression in 5 normal and 13 SCC patient samples that are quantified in Fig 2I. B) qRT-PCR analysis of B56α mRNA expression in different skin lesions graphed relative to one of the normal skin samples. BCC: Basal Cell Carcinoma, DP: Dermatofibrosarcoma Protuberans, MCC: Merkel Cell Carcinoma, MC: Mucinous Carcinoma, SK: Seborrheic Keratosis, Spindle CC: Spindle Cell Carcinoma. (TIF) [file pone.0188910.s003.tif]

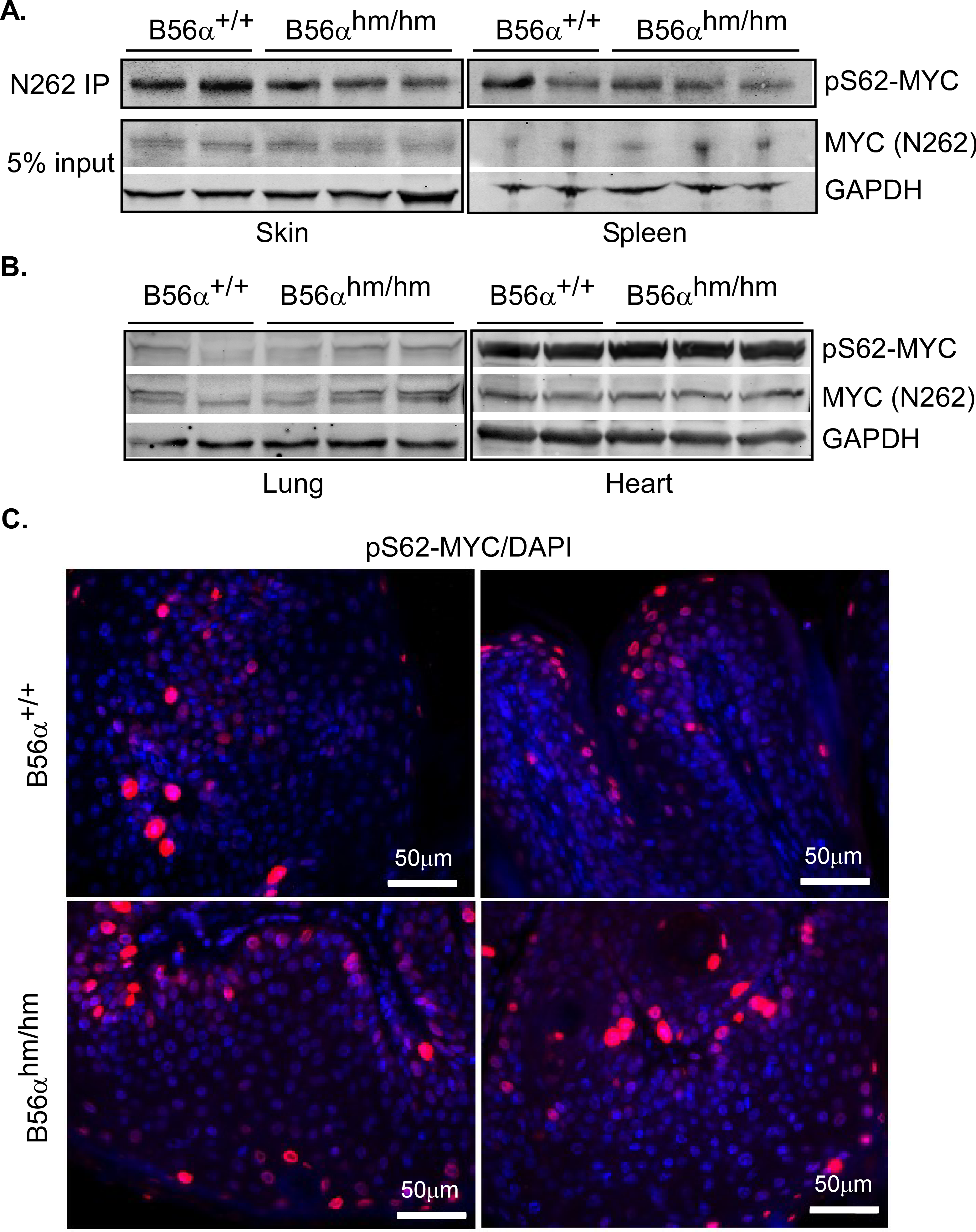

Supplement: S4 Fig — A) IP-Western of pS62-MYC from normal skin and spleen of B56α+/+ and B56αhm/hm mice. B) Western blot of pS62-MYC from normal lung and heart of B56α+/+ and B56αhm/hm mice. C) IF representative image of pS62-MYC staining (red; ab185656) of B56α+/+ and B56αhm/hm DMBA/TPA end stage papilloma lesions. DAPI (blue) is a nuclear counterstain. (TIF) [file pone.0188910.s004.tif]

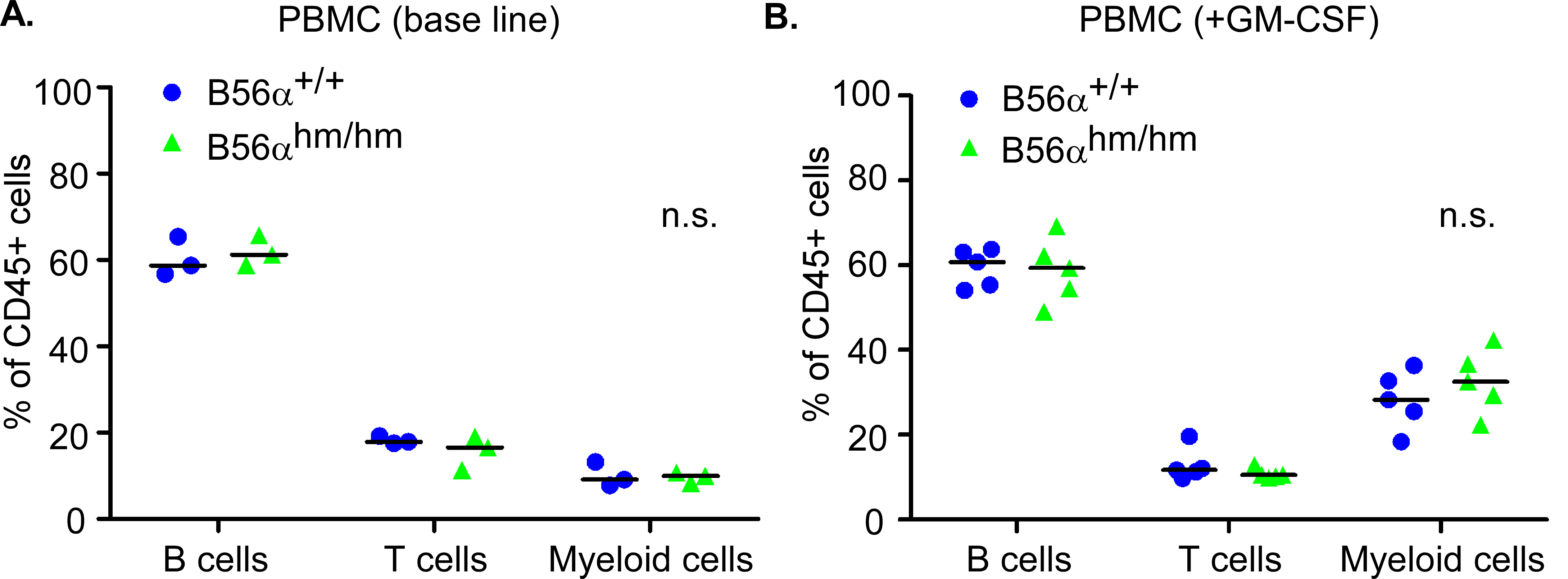

Supplement: S5 Fig — A) Flow cytometry for B cells (B220), T cells (CD3) and myeloid cells (Mac1/Gr1) within PBMCs from peripheral blood at the baseline level (n = 3 for each genotype) and after four injections with GM-CSF (n = 5 for each genotype). (TIF) [file pone.0188910.s005.tif]
