# Supplementary material for: The tumor suppressor phosphatase PP2A-B56α regulates stemness and promotes the initiation of malignancies in a novel murine model
Source: PLoS One. 2017 Nov 30;12(11):e0188910. doi: 10.1371/journal.pone.0188910 (PMC5708644; doi:10.1371/journal.pone.0188910)
Supplement: S1 Table — (PDF) [file pone.0188910.s006.pdf]

	Primer Sequence
**Forward-ex1**	5'-CGCACCAGUUGCCUGCGC-3'
**Reverse-ex1**	5'-GGCAGGGGGTGCAGCTCC-3'
**Reverse-ex3**	5'-GCCAAGAGGCCTCAAGTGTGG-3'
